# House dust mite-related respiratory allergies and probiotics: a narrative review

**DOI:** 10.1186/s12948-018-0092-9

**Published:** 2018-06-19

**Authors:** Filippo Fassio, Fabio Guagnini

**Affiliations:** 1 SOC Allergologia e Immunologica Clinica, Azienda USL Toscana Centro, Nuovo Ospedale S. Giovanni di Dio, Via di Torregalli, 3, 50143 Florence, Italy; 2 Allergy Therapeutics Italia, via IV Novembre 76, 20019 Settimo Milanese, Milan Italy

**Keywords:** House dust mite, Allergy, Probiotics

## Abstract

The socio-economic burden of allergic respiratory conditions on continental Europe is even higher than that of mainstream diseases, such as diabetes and cardiovascular disease, as allergic rhinitis alone accounts for billions of Euros in healthcare expenses across Europe. House dust mites (HDM) are one of the most common triggers behind allergic rhinitis and asthma. The role of probiotics in the treatment and prevention of some allergic conditions, such as atopic dermatitis, is already well recognized, whereas evidence about their efficacy in patients with respiratory allergies—while increasing—is still limited. Here the current evidence for the use of probiotics in patients with allergic rhinitis and/or asthma is discussed.

## Background

Allergies and asthma are health conditions that have become highly prevalent across the globe, triggering respiratory symptoms—such as runny nose and sneezing, shortness of breath, and wheezing—in affected individuals. One of the most common triggers behind such allergic reactions are the allergens of house dust mites (HDMs). HDMs, which thrive in warm and humid household conditions, are classified as Arachnids and form a very diverse class in the Animalia kingdom. While mites are associated with a wide range of pathological and parasitic conditions in plants and animals, HDMs are rather unique as they can trigger a type I hypersensitivity reaction in predisposed persons, which can lead to severe allergic manifestations [1]. The organism is microscopic in nature, approximately 0.2–0.4 mm in size, and proliferates well in the presence of skin cells and moulds, especially when conditions are moist and humid [1]. High HDM content can be detected in bedding materials, as mites feed on skin and hair, and it becomes natural that people inhale the allergen in large volumes when they sleep.

**Fig. 1:**
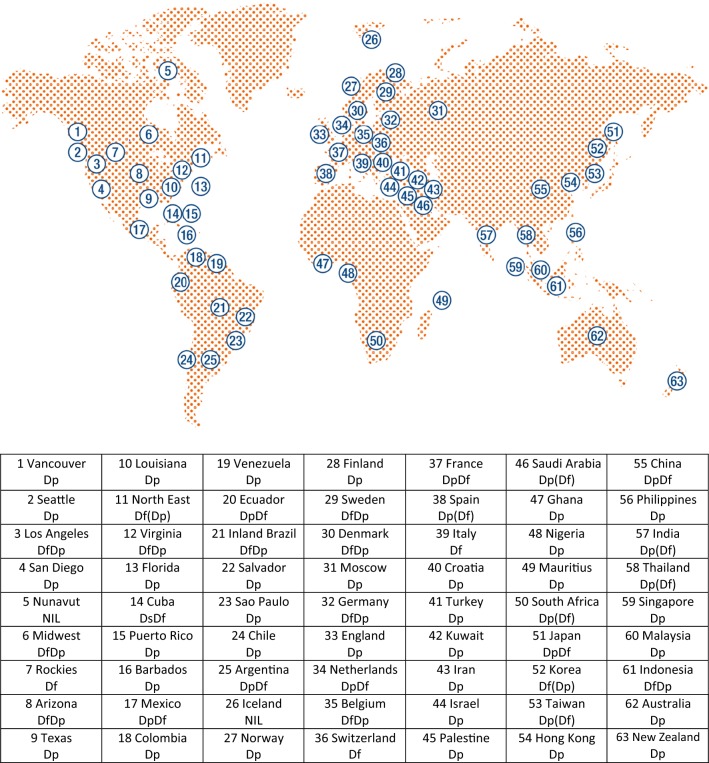
Geographical distribution of 6 subspecies of *Dermatophagoides* spp. Dp: *D. pteronyssinus* predominant; Df: *D. farinae* predominant; DpDf: mixed with more *D. pteronyssinus* than *D. farinae*; DfDp: mixed with more *D. farinae* than *D. pteronyssinus*; Ds: *D. siboney*. Parentheses indicate low levels (modified from [2])

The geographical distribution of different subspecies of HDMs is truly multi continental, as numerous research studies have indicated their prevalence across all continents except for Antarctica [2]. In regard to their ideal thriving conditions, temperatures in the range of 18–24 °C together with humidity levels of 70% and above are perfect for the organism, whereas the prevalence of various subspecies of HDMs is relatively low in environments with less than 50% relative humidity [2]. The effect of humidity on HDM proliferation is clearly visible in regions of British Columbia, Canada, in which high moisture content in the air supports high prevalence and proliferation of the organism. However, as altitude increases, and humidity levels decrease, the mite infestation rate decreases significantly. It also must be noted that altitude alone is not the only limiting factor that affects the proliferation of HDM as seen in the high-altitude regions of the Andes in South America, where the distribution of the organism is extensive [2]. Figure 1 presents a schematic representation of the distribution of 6 subspecies of HDMs across different regions of the globe [2].

## History of HDMs in medicine

HDMs belong to the taxonomic group called Astigmata and were established as a prominent source of indoor allergens with the discovery of the house mite species *Dermatophagoides pteronyssinus* in 1967 [1]. While Prof. Reindert Voorhorst is well recognized for presenting the organism to the global scientific community in that year, other prominent contributors dating back to the early 20’s and 30’s laid the foundation for research on house dust mite-related allergies in humans. Prominent examples include Prof. Willem Storm van Leeuwen, who observed that housing conditions with high moisture content was not conducive for patients suffering from asthma, and researcher Marise Spieksma-Boezeman, who identified and even cultured mite samples belonging to the genus *Dermatophagoides* in samples of house dust during her research. At that time, however, the organism had no research significance regarding its role as an allergen in humans [3].

### Epidemiology

It can be estimated that approximately 1–2% of the general population worldwide has allergic sensitization towards HDMs with significant variability depending on the cohort being studied [4]. In developed countries, this rate is much higher: in Europe, HDM allergic sensitization is above 20%; it is rising up to 40% in some cohorts in North America and to over 80% in a cohort of paediatric patients in Taiwan [4].

This result demonstrates that substantial diversity in HDM allergic sensitization prevalence exists and depends on factors such as ethnicity, socio-economic conditions, geographical variations and even the diagnostic paradigm that is adopted. For this reason, a wide range of confounding factors need to be considered to understand the epidemiology of HDM-mediated allergies [5].

A very common manifestation of HDM allergic sensitization is rhinitis, the symptoms of which include sneezing, and stuffy and running nose. These symptoms may not appear to be very serious and thus people often ignore them until the conditions become unbearable. However, a recent research article stated that the socio-economic burden of allergic rhinitis on continental Europe is even higher than that of mainstream health conditions, such as diabetes and cardiovascular disease [6]. When scaled in terms of cost to the continental economy, allergic rhinitis accounts for billions of Euros in healthcare expenses across Europe [6]. When this statistic is seen in context of the fact that allergy prevalence is rising (more than 50% of Europeans will suffer from allergic rhinitis in 10 years), the economic burden from the entire European Union’s perspective is enormous, to put it mildly [7].

### Pathogenic mechanism of HDM-related allergies

Due to the rising prevalence of different allergic conditions in recent decades, there has been a massive surge in research endeavours towards understanding their underlying pathogenic mechanisms and clinical implications [8]. Research studies have clearly shown that allergic manifestations increase significantly in individuals when there is a disruption in the T helper balance with polarization toward a Th2 phenotype, and that the dynamics of the gut microbiota has a role in maintaining this immune response balance [8]. Multiple immunological research studies have stated that a healthy gut microbiota helps in building and maintaining a well-balanced immune system in humans. Moreover, the World Health Organization has emphasized on the role of probiotic agents in enriching intestinal microflora.

A study by Calderon et al. [4] described a direct correlation between HDM-mediated allergies and the level of exposure to the organism itself during the early life of an individual. This study stated that young children in their first 3 years of life have the highest chance of contracting the allergy when exposed to HDM allergens, such as Der p 1 and Der f 1, at levels of 10 mcg/g of carpet dust. The study further added that a prior family history of allergy positively affects development of the allergy in these young children [4]. When exposure to the HDM allergens is as low as 0.1 mcg/g of dust, the probability of contracting HDM allergy is significantly reduced. While there appears to be a linear relationship between the level of exposure to allergens and HDM allergy, a wide range of scientific studies state otherwise. There are reports of low prevalence of HDM allergy in young children who are either exposed to very high or low levels of HDM allergens in their first 5 years of life. This non-linear association may be explained in terms of the high dose tolerance phenomenon that is observed in the case of cat allergens, but there is still no conclusive scientific evidence in this regard [9]. Different allergens from HDMs vary significantly in size, and this aspect can significantly impact the way that they are inhaled. Allergens that are smaller in size can penetrate deep into the lungs, but their rate of inhalation is less than that of large allergens, which can trigger an early phase immune response and elicit rather mild clinical symptoms [10]. As allergens are inhaled by the exposed individual, they enter the epithelial linings of the lungs and trigger the spread of dendritic cells into the lymph nodes. This stage is followed by the triggering of Th2 cell-mediated immunity and consequent inflammation of the lung’s epithelial linings [11]. Calderon et al. [4] further added that the efficacy of HDM allergens in eliciting a powerful allergic reaction is compounded by stimulation of the innate immune system along with the Th2 cell-mediated immune response.

### Therapeutic role of probiotics against respiratory allergies

Considering the massive socio-economic burden of different allergic conditions on global healthcare resources, there is a considerable research impetus towards finding pioneering therapeutic and preventive interventions. In this regard, the role of the gut microbiota in shaping the immune response against conditions such as HDM allergy was anticipated in a previous section. Following up, there are new findings that suggest that the dynamics of gut colonization in infants play a critical role in shaping the immune system, and there is a possibility of enriching the gut microflora using probiotics that could contribute towards the prevention of allergic conditions in later life [12, 13]. The microbiome bacteria can affect T helper responses by eliciting T helper-1 (Th1) cytokine production and/or by inducing T regulatory cells (Treg) [14], thereby suppressing Th2 responses. *Lactobacillus* bacteria affect Treg cells by generating semi-mature dendritic cells and increasing the expression of CD40; they are capable of inhibiting Interleukin (IL)-4 and IL-5 while inducing specific regulatory cytokines, such as transforming growth factor (TGF-β) and IL-10 [15]. Probiotics can also enhance local IgA production, which directly affects mucosal defences [15], and they have been involved in the maturation of adaptive T cell immunity by stimulating the production of IL-17 [16].

One of the first observations about the role of probiotic supplementation for the prevention of atopic eczema dates to 2001, when Kalliomaki et al. [17] found a significant reduction in the probability of developing atopic eczema by age 2 when supplementation of *Lactobacillus rhamnosus* was given for 2–4 weeks prenatally and 6 months postnatally; these results were confirmed in two subsequent follow-up studies in which a minor incidence of atopic disease was confirmed after 4 and after 7 years, respectively [18, 19].

A recent systematic review and meta-analysis of the literature that documented the use of probiotic agents for the prevention of allergic conditions in early life was carried out by Zuccotti et al. [12]. The authors analysed 29 RCTs on a total of 4755 children and found a significant reduction (odds ratio 0.78, CI 0.69–0.89) in the development of AD when dietary probiotic supplementation was added during pregnancy and early infancy with an even more prominent effect when a probiotic mixture was employed [12]. They concluded that the current literature demonstrates an overall benefit of probiotic supplementation for the prevention of eczema in high-risk infants. The same study further highlighted that lower diversity of the gut microbiota is associated with an increased risk of type I hypersensitivity reactions in young children [12]. It may be possible that when probiotic supplementation is given to these susceptible individuals, it results in a more favourable “allergy-protecting” gut microbiota.

In regard to the role of probiotic supplementation in patients with allergic rhinitis, Vilà-Nadal et al. [20], recently reviewed this topic [21]. Table 1 highlights different probiotic strains that have been evaluated in allergic rhinitis caused by allergens such as pollen and HDMs in adults and children.

**Table 1:** Different probiotic strains that were evaluated in patients with allergic rhinitis. Modified from [21]

Probiotic agent	Study population	N	Type of allergic rhinitis/allergen	Primary end point	Statistical significance	References
*L. acidophilus* and *B. lactis*	Children	47	Birch pollen	Symptoms score	p = 0.078	[41]
*E. coli* strain Nissle 1917	Adults	34	Grass pollen	Symptoms score	p = 0.257	[42]
*L. johnsonii* EM	Children	63	Perennial rhinitis	Symptoms score	p = 0.09	[43]
*B. longum* BB536	Adults	40	Japanese cedar pollen	Ocular symptoms score	p = 0.04	[44]
*L. paracasei* (LP-33)	Children	80	Perennial	Quality of life	p = 0.037	[45]
*L. paracasei*	Children	90	House dust mites	Quality of life	p < 0.0001	[27]
*L. paracasei* KW3110	Adults	126	Japanese cedar pollen	Symptoms score	p < 0.05	[46]
*L. paracasei* ST-11	Adults	31	Grass pollen	Nasal provocation test	Ns	[47]
*L. paracasei* NCC2461	Adults	28	Grass pollen	Nasal congestion	Ns	[48]
*L. paracasei* 33	Adults	425	Grass pollen	Rhinitis quality of life (RQLQ score)	p = 0.0255	[49]

The authors of this review conclude that probiotics appear to have beneficial therapeutic effects in allergic response as improvements in symptom scores and quality of life have been observed in these patients. In contrast, no positive impact was shown for the prevention of asthma and respiratory diseases [21].

In another systematic review on the effect of probiotics in AR, Zajac et al. [22] considered 23 randomized controlled trials on a total 1919 patients; quantitative analysis found a positive significant effect of probiotics on rhinitis-related quality of life (RQLQ Global Score and RQLQ Nose Score), whereas a positive but non-significant effect was found regarding symptom scores. Despite the lack of evidence for an improvement of symptom scores and the significant heterogeneity of these RCTs, the authors concluded that probiotics may have beneficial effects in patients affected by AR and that further studies are needed to adequately address this question [22].

It has to be underlined that probiotics effects must be considered strain-specific; therefore, future studies are required to investigate which strain(s) can be considered the most suitable in order to achieve the best immunomodulatory effect in patients affected by allergic respiratory diseases [23].

## Role of probiotics in HDM-related allergic conditions

Scientific evidence assessing the role of probiotics in the treatment of HDM-related respiratory allergies, while limited, is increasing.

The potential capacity of *Lactobacillus plantarum* NCIMB8826 to reduce the allergic response in HDM allergic patients was evaluated in vivo and in vitro. In this study [24], it was shown that the co-application of *L. plantarum* and Der p 1 induced a Th1-shift in the immune response with upregulation of IgG and Th1-cytokine (IFN-γ and IL-12) production, moderate stimulation of IL-10, reduction of bronchoalveolar lavage eosinophilia after allergen challenge and suppression of allergen-specific IgE-response.

In one study on 29 patients affected by allergic asthma and HDM sensitization, a 4-week treatment with synbiotics significantly reduced the systemic production of Th2-cytokines (namely, IL-5) after allergen challenge and improved peak expiratory flow, despite a lack of any observed effects on bronchial inflammation markers [25].

Another recent and interesting approach consists of probiotic-impregnated bedding material. In the first pilot RCT in which 20 patients with AR and HDM sensitization used such materials for 8 weeks, no differences were found in HDM allergen levels compared to the placebo, whereas a significant improvement was reported by patients in terms of rhinitis-related symptom reduction and quality of life [26].

Of interest, it has been shown that even heat-killed lactic acid bacteria can be of help in the treatment of patients with respiratory allergy and HDM sensitization. Peng et al. [27] showed that heat-killed *Lactobacillus paracasei* supplementation significantly improved rhinitis-related quality of life scores in a group of 90 children with HDM sensitization.

The therapeutic potential of probiotic strains against allergic conditions was also explored by an observational study published in 2014. This study focused on the efficacy of two different symbiotic formulations (*Lactobacillus acidophilus* NCFM, *Bifidobacterium lactis* BL-04 and fructo-oligosaccharide (FOS); *Lactobacillus plantarum* LP01, *Lactobacillus paracasei* LPC00 and FOS) in the management of seasonal allergic rhinitis (SAR) and perennial allergic rhinitis (PAR) [28]. Aspects such as total nasal symptoms score, ARIA classification [29] for AR severity and data on the concomitant treatments were collected at the beginning of the study and evaluated at two different time points, 1 and 2 months after the beginning of the supplementation period. The results of this study highlighted a decrease in the total nasal symptoms score and an improvement in the AR severity according to the ARIA classification of rhinitis at both the time points. The reduction in the symptom score was statistically significant for the probiotic combination of *Lactobacillus plantarum* and *Lactobacillus paracasei* in patients with PAR, whereas the symbiotic combination against SAR showed a statistically significant improvement at only the first timepoint. However, a concomitant decrease in the consumption of symptomatic medications, such as antihistamine, and corticosteroids drugs significantly and progressively decreased after 2 and 4 months of treatment.

The observed decrease in the utilization of co-administered oral antihistamine drugs and oral corticosteroid drugs during the study period with this symbiotic multistrain combination may represent additional evidence of the beneficial effect of probiotics in the management of allergic rhinitis [28].

As it has already been mentioned before, identification of the most suitable probiotic strains in order to achieve a significant immunomodulatory effect in patients with HDM-related allergic conditions, will be one main challenge in this research field in the near future. Moreover, more research and effort are needed to improve the formulation of probiotic products (e.g. resistance to gastrointestinal conditions; drops—especially for pediatric use) to assure higher efficacy.

## Future perspectives on the role of probiotics as adjuvants in allergen immunotherapy

Allergen-specific immunotherapy (AIT) is a well-established treatment option for allergic rhinitis, including HDM-triggered AR [30]. AIT triggers immunoregulatory mechanisms, including Treg cell activation, leading to the suppression of Th2 responses in favour of tolerance towards the allergen [31].

Adjuvants have been shown to amplify the effect of AIT by modulating the immune response to this therapy, thus increasing both its efficacy and safety [32].

In recent times, it has been hypothesized that probiotics, which stimulate innate immunity via toll-like receptor activation, could be used as adjuvants in AIT.

The effectiveness of probiotics as potential adjuvants in sublingual AIT (SLIT) has been studied in animal models; multiple lactic acid bacteria strains were found to act as Th1/possibly Treg inducers, and therefore—in the authors’ opinion—they could represent valid candidates for adjuvants in SLIT [33].

In a murine model of mite allergy, immunotherapy with a recombinant Der p 1 allergen plus a probiotic strain (*E. coli* Nissle 1917) prevented the development of allergic symptoms after an airway challenge with mite extracts; moreover, it was observed that it contributed to the upregulation of allergen-specific IgG2a production, down-regulation of specific IgE production and induction of a strong reduction of IL-5 secretion by allergen-restimulated splenocytes [34].

In such preclinical models, probiotics acting as pure Th1 adjuvants did not seem to improve the efficacy of SLIT, whereas probiotic strains acting as Th1/Treg adjuvants (stimulating both IL-12 and IL-10 secretion by DCs) significantly enhanced SLIT efficacy [35].

In a study by Jerzynska et al. [36] on children with AR and grass sensitization, the addition of probiotics (*Lactobacillus rhamnosus* GG) to SLIT improved symptom scores and the induction of T regulatory cells after 5 months of treatment relative to children treated with SLIT alone or placebo.

In one study, *Lactobacillus acidophilus* and *Bifidobacterium lactis* contributed to improved asthma control test (ACT) scores in children with allergic asthma undergoing AIT for HDM; the effect was additive relative to the sole effect of AIT [37].

*Clostridium butyricum* coadministration with AIT has shown to improve the efficacy of AIT in patients with AR and asthma [38, 39]; this effect has been attributed to an increase in allergen-specific regulatory B cells.

Recently, in an observational real-life study, Rossi et al. [40] showed that supplementation with a symbiotic (consisting of *Lactobacillus rhamnosus*, *Bifidobacterium lactis* and FOS) during AIT for AR may act synergistically to improve the clinical efficacy of sublingual allergen immunotherapy. After 2 and 4 months of treatment, patients who were administered AIT plus probiotics showed a trend towards the reduction of symptoms scores together with a significant improvement in medication scores, number of “well days”; and no relevant local or systemic adverse reactions [40].

## Conclusion

The use of probiotics in the treatment of allergic diseases is very promising, thanks to increasing evidence of their immunomodulatory effects. Their role in the treatment and prevention of some allergic conditions, such as atopic dermatitis, is already well recognized, whereas evidence about their efficacy in patients with respiratory allergies—while increasing—is still limited.

The prospective of employing probiotics as novel adjuvants for AIT is intriguing and already supported by a number of interesting studies.

Future research is needed to improve our understanding of the use of probiotics in the treatment of allergic conditions and as an adjuvant in immunotherapy; in particular, we need to better understand which probiotic strains are the most suitable for these purposes and which immunologic mechanisms are involved. Moreover, evidence is still lacking regarding which formulations will guarantee the best results, what are the best timing and duration for probiotic administration in order to prevent/modulate HDM-related allergic conditions, and if a critical dose (beside the 10^9^ colony forming units generally considered) exists.

## References

[1] Portnoy J, Miller JD, Williams PB, Chew GL, Miller JD, Zaitoun F (2013). Environmental assessment and exposure control of dust mites: a practice parameter. Ann Allergy Asthma Immunol.

[2] Thomas WR (2010). Geography of house dust mite allergens. Asian Pac J Allergy Immunol.

[3] Spieksma FT, Dieges PH (2004). The history of the finding of the house dust mite. J Allergy Clin Immunol.

[4] Calderón MA, Linneberg A, Kleine-Tebbe J, De Blay F, De Rojas DHF, Virchow JC (2015). Respiratory allergy caused by house dust mites: what do we really know?. J Allergy Clin Immunol.

[5] Bousquet PJ, Chinn S, Janson C, Kogevinas M, Burney P, Jarvis D (2007). Geographical variation in the prevalence of positive skin tests to environmental aeroallergens in the European community respiratory health survey I. Allergy.

[6] Rossi O, Massaro I, Caminati M, Quecchia C, Fassio F, Heffler E (2015). Escaping the trap of allergic rhinitis. Clin Mol Allergy.

[7] Calderon MA, Demoly P, Gerth van Wijk R, Bousquet J, Sheikh A, Frew A (2012). EAACI: a European declaration on immunotherapy. Designing the future of allergen specific immunotherapy. Clin Transl Allergy.

[8] Fassio F, Guagnini F (2016). Probiotics, gut microbiota and immunomodulation: is this the key to counteract the allergy epidemics?. J Pharm Nutr Sci.

[9] Platts-Mills T, Vaughan J, Squillace S, Woodfolk J, Sporik R (2001). Sensitisation, asthma, and a modified Th2 response in children exposed to cat allergen: a population-based cross-sectional study. Lancet.

[10] Custovic A, Woodcock H, Craven M, Hassall R, Hadley E, Simpson A (1999). Dust mite allergens are carried on not only large particles. Pediatr Allergy Immunol.

[11] Lambrecht BN, Hammad H (2010). The role of dendritic and epithelial cells as master regulators of allergic airway inflammation. Lancet.

[12] Zuccotti G, Meneghin F, Aceti A, Barone G, Callegari ML, Di Mauro A (2015). Probiotics for prevention of atopic diseases in infants: systematic review and meta-analysis. Allergy.

[13] Ismail IH, Licciardi PV, Tang MLK (2013). Probiotic effects in allergic disease. J Paediatr Child Health.

[14] Smith-Norowitz TA, Bluth MH (2016). Probiotics and diseases of altered IgE regulation: a short review. J Immunotoxicol.

[15] Campeotto F, Suau A, Kapel N, Magne F, Viallon V, Ferraris L (2011). A fermented formula in pre-term infants: clinical tolerance, gut microbiota, down-regulation of faecal calprotectin and up-regulation of faecal secretory IgA. Br J Nutr.

[16] Isailovic N, Daigo K, Mantovani A, Selmi C (2015). Interleukin-17 and innate immunity in infections and chronic inflammation. J Autoimmun.

[17] Kalliomäki M, Salminen S, Arvilommi H, Kero P, Koskinen P, Isolauri E (2001). Probiotics in primary prevention of atopic disease: a randomised placebo-controlled trial. Lancet.

[18] Kalliomäki M, Salminen S, Poussa T, Arvilommi H, Isolauri E (2003). Probiotics and prevention of atopic disease: 4-year follow-up of a randomised placebo-controlled trial. Lancet.

[19] Kalliomäki M, Salminen S, Poussa T, Isolauri E (2007). Probiotics during the first 7 years of life: a cumulative risk reduction of eczema in a randomized, placebo-controlled trial. J Allergy Clin Immunol.

[20] Vilà-Nadal G, Phillips-Anglés E, Domínguez-Ortega J (2016). The use of probiotics in respiratory allergy. J Pharm Nutr Sci..

[21] Domínguez-Ortega J, Phillips-Anglés E (2016). The use of probiotics in respiratory allergy. J Pharm Nutr Sci.

[22] Zajac AE, Adams AS, Turner JH (2015). A systematic review and meta-analysis of probiotics for the treatment of allergic rhinitis. Int Forum Allergy Rhinol.

[23] Toscano M, De Grandi R, Pastorelli L, Vecchi M, Drago L (2017). A consumer’s guide for probiotics: 10 golden rules for a correct use. Dig Liver Dis.

[24] Hisbergues M, Magi M, Rigaux P, Steuve J, Garcia L, Goudercourt D (2007). In vivo and in vitro immunomodulation of der p 1 allergen-specific response by *Lactobacillus plantarum* bacteria. Clin Exp Allergy.

[25] Van De Pol MA, Lutter R, Smids BS, Weersink EJM, Van Der Zee JS (2011). Synbiotics reduce allergen-induced T-helper 2 response and improve peak expiratory flow in allergic asthmatics. Allergy.

[26] Berings M, Jult A, Vermeulen H, De Ruyck N, Derycke L, Ucar H (2017). Probiotics-impregnated bedding covers for house dust mite allergic rhinitis: a pilot randomized clinical trial. Clin Exp Allergy.

[27] Peng GC, Hsu CH (2005). The efficacy and safety of heat-killed *Lactobacillus paracasei* for treatment of perennial allergic rhinitis induced by house-dust mite. Pediatr Allergy Immunol.

[28] Manzotti G, Heffler E, Fassio F (2014). Multi-strain symbiotic preparations as a novel adjuvant approach to allergic rhinitis. JCI.

[29] Brozek JL, Bousquet J, Baena-Cagnani CE, Bonini S, Canonica GW, Casale TB (2010). Allergic rhinitis and its impact on asthma (ARIA) guidelines: 2010 revision. J Allergy Clin Immunol.

[30] Eifan AO, Calderon MA, Durham SR (2013). Allergen immunotherapy for house dust mite: clinical efficacy and immunological mechanisms in allergic rhinitis and asthma. Expert Opin Biol Ther.

[31] Fujita H, Meyer N, Akdis M, Akdis CA (2012). Mechanisms of immune tolerance to allergens. Chem Immunol Allergy.

[32] Pfaar O, Cazan D, Klimek L, Larenas-Linnemann D, Calderon MA (2012). Adjuvants for immunotherapy. Curr Opin Allergy Clin Immunol.

[33] Van Overtvelt L, Moussu H, Horiot S, Samson S, Lombardi V, Mascarell L (2010). Lactic acid bacteria as adjuvants for sublingual allergy vaccines. Vaccine.

[34] Adam E, Delbrassine L, Bouillot C, Reynders V, Mailleux AC, Muraille E (2010). Probiotic *Escherichia coli* Nissle 1917 activates DC and prevents house dust mite allergy through a TLR4-dependent pathway. Eur J Immunol.

[35] Moingeon P, Lombardi V, Baron-Bodo V, Mascarell L (2017). Enhancing allergen-presentation platforms for sublingual immunotherapy. J Allergy Clin Immunol Pract.

[36] Jerzynska J, Stelmach W, Balcerak J, Woicka-Kolejwa K, Rychlik B, Blauz A (2016). Effect of Lactobacillus rhamnosus GG and vitamin D supplementation on the immunologic effectiveness of grass-specific sublingual immunotherapy in children with allergy. Allergy Asthma Proc.

[37] Kardani AK, Fitri LE, Barlianto W, Olivianto E, Kusuma C (2013). The effect of house dust mite immunotherapy, probiotic and *Nigella sativa* in the number of Th17 cell and asthma control test score. IOSR J Dent Med Sci.

[38] Liu J, Chen FH, Qiu SQ, Yang LT, Zhang HP, Liu JQ (2016). Probiotics enhance the effect of allergy immunotherapy on regulating antigen specific B cell activity in asthma patients. Am J Transl Res.

[39] Xu LZ, Yang LT, Qiu SQ, Yang G, Luo XQ, Miao BP (2016). Combination of specific allergen and probiotics induces specific regulatory B cells and enhances specific immunotherapy effect on allergic rhinitis. Oncotarget.

[40] Rossi R, Monasterolo G (2016). Combination of probiotics and sublingual immunotherapy in allergic rhinitis: a real-life study. J Pharm Nutr Sci.

[41] Ouwehand AC, Nermes M, Collado MC, Rautonen N, Salminen S, Isolauri E (2009). Specific probiotics alleviate allergic rhinitis during the birch pollen season. World J Gastroenterol.

[42] Dölle S, Berg J, Rasche C, Worm M (2014). Tolerability and clinical outcome of coseasonal treatment with *Escherichia coli* strain Nissle 1917 in grass pollen-allergic subjects. Int Arch Allergy Immunol.

[43] Lue KH, Sun HL, Lu KH, Ku MS, Sheu JN, Chan CH (2012). A trial of adding *Lactobacillus johnsonii* EM1 to levocetirizine for treatment of perennial allergic rhinitis in children aged 7–12 years. Int J Pediatr Otorhinolaryngol.

[44] Xiao JZ, Kondo S, Yanagisawa N, Takahashi N, Odamaki T, Iwabuchi N (2006). Effect of probiotic *Bifidobacterium longum* BB536 [corrected] in relieving clinical symptoms and modulating plasma cytokine levels of Japanese cedar pollinosis during the pollen season. A randomized double-blind, placebo-controlled trial. J Investig Allergol Clin Immunol.

[45] Wang MF, Lin HC, Wang YY, Hsu CH (2004). Treatment of perennial allergic rhinitis with lactic acid bacteria. Pediatr Allergy Immunol.

[46] Yonekura S, Okamoto Y, Okawa T, Hisamitsu M, Chazono H, Kobayashi K (2009). Effects of daily intake of *Lactobacillus paracasei* strain KW3110 on Japanese cedar pollinosis. Allergy Asthma Proc.

[47] Wassenberg J, Nutten S, Audran R, Barbier N, Aubert V, Moulin J (2011). Effect of *Lactobacillus paracasei* ST11 on a nasal provocation test with grass pollen in allergic rhinitis. Clin Exp Allergy.

[48] Perrin Y, Nutten S, Audran R, Berger B, Bibiloni R, Wassenberg J (2014). Comparison of two oral probiotic preparations in a randomized crossover trial highlights a potentially beneficial effect of *Lactobacillus paracasei* NCC2461 in patients with allergic rhinitis. Clin Transl Allergy.

[49] Costa DJ, Marteau P, Amouyal M, Poulsen LK, Hamelmann E, Cazaubiel M (2014). Efficacy and safety of the probiotic *Lactobacillus paracasei* LP-33 in allergic rhinitis: a double-blind, randomized, placebo-controlled trial (GA2LEN study). Eur J Clin Nutr.

